# Proteome-wide analysis of human disease mutations in short linear motifs: neglected players in cancer?†
†Electronic supplementary information (ESI) available: Supplementary files 1–22 and supplementary Fig. 1–3. See DOI: 10.1039/c4mb00290c



**DOI:** 10.1039/c4mb00290c

**Published:** 2014-07-24

**Authors:** Bora Uyar, Robert J. Weatheritt, Holger Dinkel, Norman E. Davey, Toby J. Gibson

**Affiliations:** a Structural and Computational Biology Unit , European Molecular Biology Laboratory , Meyerhofstrasse 1 , 69117 , Heidelberg , Germany . Email: bora.uyar@embl.de ; Email: toby.gibson@embl.de; b MRC Laboratory of Molecular Biology , Francis Crick Avenue , Hills Road , Cambridge CB2 0QH , UK; c Banting and Best Department of Medical Research and Donnelly Centre , University of Toronto , Toronto , Ontario M5S 3E1 , Canada; d Department of Physiology , University of California, San Francisco , San Francisco , California , USA

## Abstract

Disease mutations are traditionally thought to impair protein functionality by disrupting the folded globular structure of proteins. However, 22% of human disease mutations occur in natively unstructured segments of proteins known as intrinsically disordered regions (IDRs). This therefore implicates defective IDR functionality in various human diseases including cancer. The functionality of IDRs is partly attributable to short linear motifs (SLiMs), but it remains an open question how much defects in SLiMs contribute to human diseases. A proteome-wide comparison of the distribution of missense mutations from disease and non-disease mutation datasets revealed that, in IDRs, disease mutations are more likely to occur within SLiMs than neutral missense mutations. Moreover, compared to neutral missense mutations, disease mutations more frequently impact functionally important residues of SLiMs, cause changes in the physicochemical properties of SLiMs, and disrupt more SLiM-mediated interactions. Analysis of these mutations resulted in a comprehensive list of experimentally validated or predicted SLiMs disrupted in disease. Furthermore, this in-depth analysis suggests that ‘prostate cancer pathway’ is particularly enriched for proteins with disease-related SLiMs. The contribution of mutations in SLiMs to disease may currently appear small when compared to mutations in globular domains. However, our analysis of mutations in predicted SLiMs suggests that this contribution might be more substantial. Therefore, when analysing the functional impact of mutations on proteins, SLiMs in proteins should not be neglected. Our results suggest that an increased focus on SLiMs in the coming decades will improve our understanding of human diseases and aid in the development of targeted treatments.

## Abstract 2

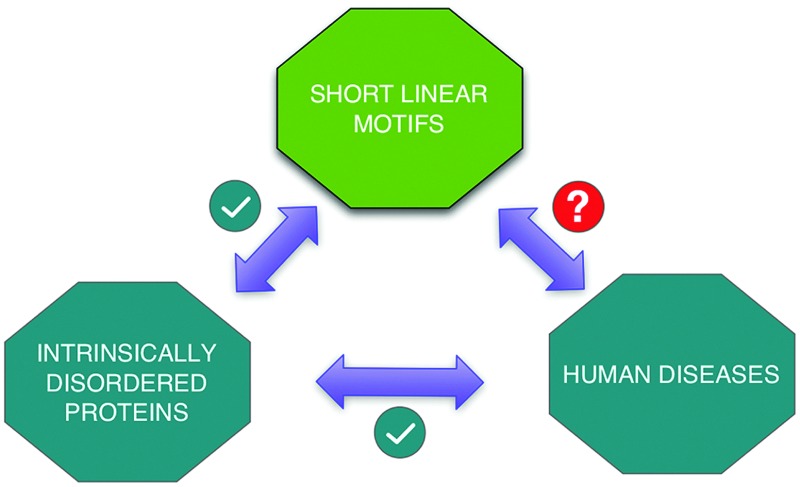
Mutations in short linear motifs impair the functions of intrinsically disordered proteins in cellular signaling/regulation and contribute substantially to human diseases.

## Introduction

Alterations of the human genome are the source of many diseases including cancer. Such alterations can be rare at microscopic levels (*e.g.* aneuploidies, chromosomal rearrangements), frequent at sub-microscopic levels (*e.g.* insertions, deletions, inversions, duplications, copy number variations) and most typical as single nucleotide substitutions.^1^ Single nucleotide substitutions within the protein coding regions of the genome can hit splice sites, shift the reading frame of a gene, or introduce stop codons. Substitutions that change amino acids of the protein product, known as ‘missense mutations’, can have adverse effects on protein structure and function. Most of the disease-related missense mutations (∼78%) are found within ordered/globular, structured regions of proteins,^2^ in particular, regions of low solvent accessibility.^3^ Such mutations in the globular domains may impact the stability and folding of the domains,^4^ impair active sites^5^ or alter binding pockets.^6^

Many proteins contain functionally important regions that lack stable tertiary structures in solution, known as intrinsically disordered regions (IDRs).^7–12^ Although disease-related missense mutations are enriched in ordered regions,^13^ they can also have an impact on the functionally important regions of IDRs.^2,14^ For instance, proteome-wide analyses of disease-related mutations have shown that gain or loss of post-translational modification sites, which are generally found in IDRs, contributes to human diseases.^15–17^ Moreover, IDRs are enriched in proteins implicated in human diseases,^18^ for instance, 80% of human cancer-associated proteins contain extensive IDRs.^19^

IDRs are frequently observed in the human proteome. A significant proportion of the human proteome is disordered (∼22% of all the residues) and ∼35% of the proteins contain at least one disordered segment longer than 30 residues.^20^ IDR-containing proteins, often referred to as intrinsically disordered proteins (IDPs), are core components of the cellular machinery and are particularly associated with transcription, translation, signal transduction, and the cell cycle.^21^ Depending on the interaction partner and the intra-cellular context, IDPs can take various conformations. Thus, IDPs are able to mediate multiple signaling events^21–24^ and serve as hubs in protein–protein interaction networks.^25^

A key class of protein interaction modules predominantly found within IDRs is the short linear motifs (SLiMs),^26^ which are short (3–10 amino acids long) peptide segments of proteins.^27^ SLiMs can serve as sites of proteolytic cleavage, post-translational modification, ligand binding or ligand docking, or as signals for sub-cellular targeting or proteasomal degradation.^28^ This wide functional spectrum is achieved by recognition of SLiMs by various classes of protein globular domains. As opposed to globular domains, SLiMs take up a very small sequence space. Consequently, IDRs can be densely packed with multiple SLiMs, which can sometimes overlap and act as regulatory switches.^29,30^

**Fig. 1:**
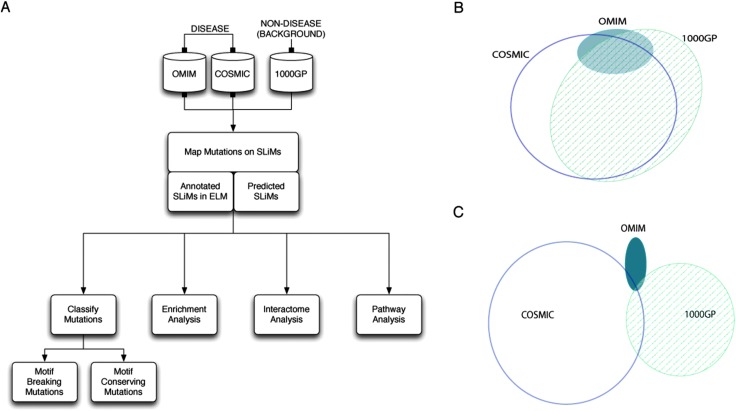
Analysis of mutations in short linear motifs. (A) Pipeline for the analysis of mutations in SLiMs. (B) Proteins shared by mutation datasets (OMIM: Inherited Disease Mutations from UniProt, COSMIC: Catalog of Somatic Mutations in Cancer, 1000GP: missense mutations from the 1000 Genomes Project). (C) Mutated sites shared by mutation datasets.

With the exception of post-translational modification sites,^15–17^ the impact of disease-related mutations on SLiMs and the association of SLiMs with human diseases have not been studied at a proteome-wide scale with a specific focus on SLiMs. One of the previous notable studies has provided a literature review of the disease-related mutations in SLiMs.^31^ Another study has investigated whether mutations in IDRs that shift the disordered state of a residue into an ordered state (called disorder-to-order transition mutations) are enriched in experimentally validated SLiMs.^2^ However, no significant enrichment of disorder-to-order transition mutations was observed for disease-related mutations compared to neutral missense mutations. In this work, we report a proteome-wide analysis of disease-related mutations with a specific focus on SLiMs. We utilise the growing knowledge of disease and non-disease mutations generated by high-throughput sequencing and compiled by resources such as the “Catalog of Somatic Mutations In Cancer” (COSMIC)^32^ and the “1000 Genomes Project” (1000GP).^33^ We complement our analysis by mutation data annotated in UniProt^34^ for inherited human diseases compiled by “Online Mendelian Inheritance in Man” (OMIM).^35^ By comparing the distribution of disease and non-disease mutation datasets, we show that disease-related mutations are enriched in SLiMs in IDRs and they occur more frequently at functionally important residues of SLiMs. Also, in the context of protein interaction networks, we show that the number of interactions mediated by a SLiM correlates with the likelihood that a mutation affecting that SLiM will be disease-related. Based on these analyses, we report a comprehensive list of experimentally validated and predicted disease-related SLiMs. This list reveals that ‘KEGG human prostate cancer pathway’ is the pathway most enriched for proteins containing cancer-related SLiMs (see the analysis pipeline in Fig. 1A).

## Results

### Comparison of the mutation datasets

In this study, we compare the distribution of inherited disease-related missense mutations from the OMIM dataset (19 630 mutated sites in 1941 proteins) and cancer-associated somatic missense mutations from the COSMIC dataset (440 266 mutated sites in 13 941 proteins) with missense mutations from the 1000GP dataset (207 720 mutated sites in 12 755 proteins) that are assumed to have a “neutral” impact on protein structure and function (see Methods) (ESI,† Tables S1–S3). The majority of the proteins from the disease-related mutation datasets contain at least one neutral mutation (81.5% of OMIM proteins and 70.8% of COSMIC proteins are shared with the 1000GP dataset) (Fig. 1B). Conversely, the overlap between the mutated sites from disease-related mutation datasets and the neutral mutation dataset is low (5.6% of mutated sites from the OMIM dataset and 4.0% of mutated sites from the COSMIC dataset are shared with the 1000GP dataset) (Fig. 1C) (Table 1). This suggests that there are important differences to be observed between the positional distributions of the mutations within the proteins shared by different datasets.

**Table 1:** Disease-related mutation datasets (OMIM and COSMIC) and their overlap with the neutral mutation dataset (1000GP)

			Overlap with 1000GP	
Dataset	Proteins	Mutated sites	Proteins	Mutated sites
1000GP	12 755	207 720	—	—
OMIM	1941	19 630	1580 (81.5%)	1100 (5.6%)
COSMIC	13 941	440 266	9873 (70.8%)	17 705 (4.0%)

### Mutations in experimentally validated SLiMs

**Fig. 2:**
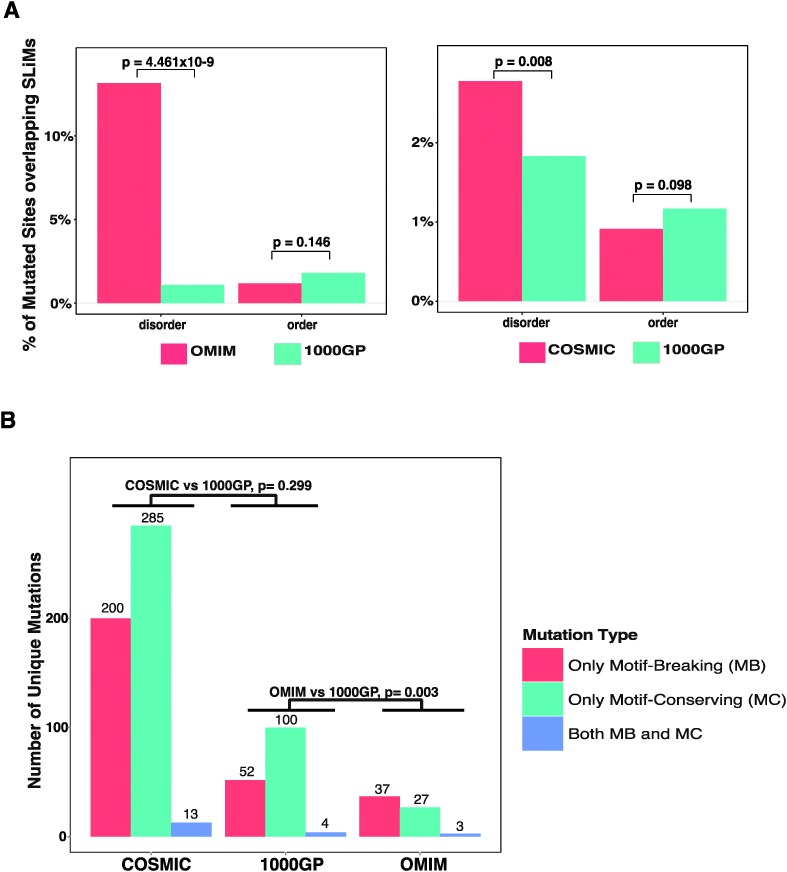
Analysis of missense mutations in experimentally validated SLiMs. (A) A site-based analysis of the enrichment of disease-related missense mutations (OMIM and COSMIC) compared to neutral missense mutations (1000GP) in ordered and disordered regions. For each comparison, SLiMs and mutated sites in the shared proteins between the compared datasets are divided into two groups as ‘disordered’ and ‘ordered’. The percentages of mutated sites overlapping the SLiMs in the respective regions are compared between OMIM and 1000GP (first panel) and COSMIC and 1000GP (second panel). (B) Classification of unique mutations overlapping the SLiMs as ‘only motif-breaking’ (MB), ‘only motif-conserving’ (MC), or ‘both MB and MC’ (see Methods).

The Eukaryotic Linear Motif (ELM) resource^28^ is a collection of experimentally validated SLiMs manually curated from the literature for eukaryotic species. The ELM resource, as of October 2013, contained a compilation of 1262 human SLiM instances for 726 proteins (ESI,† Table S4) classified into 161 classes and 6 functional types (Table 2). In total, 1262 SLiMs contain 8470 amino acid residues (average SLiM length is ∼6.7 amino acids). The database of UniProt protein sequences (see Methods) contained 19 991 proteins and had a total length of 11 140 525 amino acids (ESI,† Table S5). Thus, experimentally validated SLiM instances are found in ∼3.6% of the human proteins and they take up ∼0.08% of the residues within human protein sequences. Thus, the probability of a mutation to occur in an experimentally validated SLiM is low. After mapping the mutations from the 1000GP, the OMIM, and the COSMIC datasets onto the experimentally validated SLiMs, we observed that a small proportion of the mutations overlap SLiMs. 152 (or 0.073%) of the mutated sites from the 1000GP dataset, 53 (or 0.270%) of the mutated sites from the OMIM dataset, and 405 (or 0.092%) of the mutated sites from the COSMIC dataset overlap the experimentally validated SLiMs. Of note, disease-related missense mutation datasets show a slightly higher overlap with SLiMs than the neutral missense mutation dataset. In order to observe if there is a significant difference in the amount of overlap with SLiMs between disease-related and neutral missense mutation datasets, a pairwise comparison of the datasets (OMIM *vs.* 1000GP and COSMIC *vs.* 1000GP) was carried out (Fig. 2A). Both the mutated sites and the SLiM instances were split into two separate bins according to whether they are in ordered or disordered regions. When comparing each individual disease-related mutation dataset with the 1000GP dataset, only proteins that exist in both the respective disease-related mutation dataset and the 1000GP dataset were considered. The reason to only consider shared proteins was to avoid potential biases in terms of the order–disorder content of the non-shared proteins between the compared datasets. When considering the ordered regions of the proteome, the percentage of the mutated sites overlapping the experimentally validated SLiMs was less, although not significantly, for both the COSMIC dataset (Fisher's exact test, *p* = 0.098) and the OMIM dataset (Fisher's exact test, *p* = 0.146) than for the 1000GP dataset. This result suggests that, in the ordered regions, disease-related mutations are not enriched in experimentally validated SLiMs and even show a trend towards depletion compared to neutral missense mutations, probably because the mutations in the ordered regions are more detrimental to the protein when they hit a globular domain than a SLiM that is found in an ordered region. On the other hand, when considering only the disordered regions, a significant enrichment of disease-related mutated sites overlapping the experimentally validated SLiMs was observed for both the OMIM (Fisher's exact test, *p* = 4.461 × 10^–9^) and the COSMIC datasets (Fisher's exact test, *p* = 0.008) compared to the 1000GP dataset. Thus, a mutation in a disordered region is more likely to be disease-associated than to have no impact if the amino acid is part of a functional SLiM. It is important to note that inherited disease mutations from the OMIM dataset show a more direct causality in terms of impairing the SLiMs compared to mutations from the COSMIC dataset. This may be the consequence of higher quality annotation of OMIM mutations, which are experimentally validated to contribute to disease. Additionally, the OMIM dataset may display an acquisition bias as it contains disease-related mutations that have led to the discovery of a functional SLiM. Conversely, mutations from the COSMIC dataset should not suffer from an acquisition bias because a large portion of the COSMIC dataset is generated *via* whole genome sequencing of tumour samples.^32^ Furthermore, the majority of mutations in the COSMIC dataset lack experimental evidence for a role as cancer-drivers. Consequently, many of these will be passenger mutations that do not contribute to cancer but instead accumulate during cell proliferation.

**Table 2:** Annotated SLiMs in the ELM resource

SLiM type	Instance count	%
LIG	582	46.1
MOD	322	25.5
DOC	140	11.1
TRG	115	9.1
DEG	63	5.0
CLV	40	3.2
Total	1262	100.0

### Motif-breaking and motif-conserving mutations

Different positions within a SLiM instance have different contributions to the strength of the affinity of binding. The complementarity of the SLiM residues to the binding pocket on the interaction partner is the major constraint for the definition of the motif patterns. These patterns are represented as regular expressions that reflect the conservation pattern of each position of a motif in both the convergently evolved instances in unrelated proteins and evolutionarily conserved instances in the orthologous proteins. Thus, the functional impact of a mutation in a SLiM depends on the position of the mutated site within the SLiM and different positions of SLiMs are permissive to mutations at different levels.^27^ For example, a RGD motif (recognised by integrins) is defined exclusively by the amino acids arginine, glycine, and aspartic acid in its three positions. Any mutation in this sequence can lead to mis-recognition of the motif by the integrins. Such a mutation, which hereby is called a ‘motif-breaking’ mutation, impairs the motif functionality. On the other hand, a STAT5 Src homology 2 (SH2) domain binding motif (_p_Y[VLTFIC]xx, where ‘x’ can be any amino acid) contains one degenerately defined position ([VLTFIC] in the second position of the motif), where mutations between any of the amino acids including valine, leucine, threonine, phenyl alanine, isoleucine, and cysteine are permitted and would not impair the motif functionality. This motif also contains two wild-card positions (third and fourth position of the motif), where any mutation is permitted. Such mutations that either occur at a wild-card position or occur at a degenerately defined position within the restriction of the permitted amino acids for that position are hereby called ‘motif-conserving’ mutations. Additionally, a mutation may be classified as both ‘motif-breaking’ and ‘motif-conserving’ in the cases when the mutation affects overlapping SLiM instances (see Methods). In order to observe if mutations that hit the experimentally validated SLiMs are differently distributed within the SLiMs, the functional impact of the mutations was classified and compared. For each mutation dataset, the mutations that overlap experimentally validated SLiMs were classified as ‘only motif-breaking’, ‘only motif-conserving’, or ‘both motif-breaking and motif-conserving’ (see Table 3). The ratio of mutations exclusively classified as ‘motif-breaking’ from the 1000GP dataset (33.3%) was smaller than that from both the OMIM dataset (55.2%) and the COSMIC dataset (40.2%) (Fig. 2B) (ESI,† Tables S6–S8). The difference was significant between the OMIM dataset and the 1000GP dataset (Fisher's exact test, *p* = 0.003), while the difference between the COSMIC dataset and the 1000GP dataset was not significant (Fisher's exact test, *p* = 0.299). This result suggests that disease-related missense mutations tend to impact functionally important residues of experimentally validated SLiMs more often than neutral missense mutations. Moreover, inherited disease mutations from the OMIM dataset were significantly more frequently classified as ‘motif-breaking’ compared to the mutations from the COSMIC dataset (Fisher's exact test, *p* = 0.023). This result further emphasises the differences between the quality of the OMIM dataset and the COSMIC dataset in terms of the direct causality of the annotated mutations in human diseases.

**Table 3:** Motif-breaking and motif-conserving mutations in experimentally validated SLiM instances

Dataset	SLiMs with mutations	Mutations in SLiMs	Only motif-breaking		Only motif-conserving		Both	
			*N*	%	*N*	%	*N*	%
1000GP	144	156	52	33.3	100	64.1	4	2.6
OMIM	30	67	37	55.2	27	40.3	3	4.5
COSMIC	299	498	200	40.2	285	57.2	13	2.6

### Impact of mutations on the amino-acid properties of SLiMs

In molecular recognition, physicochemical properties of amino acids (*e.g.* charge, hydropathy, polarity, volume, chemical characteristics, hydrogen donor/acceptor availability) at the interaction interfaces are important determinants of the nature of the interaction. The physicochemical properties of amino acids in the SLiMs are reflected in the defined patterns of SLiMs.^27^ For instance, while most of the residues of nuclear localization signals favour positively charged amino acids (such as arginine and lysine), some motif classes such as degrons or 14-3-3 binding motifs require amino acids that have hydroxyl groups in the side chains (such as serine and threonine) so that the motif can be regulated *via* phosphorylation. Amino acid substitutions due to missense mutations may cause changes in the physicochemical properties of a SLiM and lead to defects in molecular recognition. For instance, R105A and R106S mutations in the nuclear localization signal of ceramide kinase-like protein cause a shift from positively charged arginine residues to uncharged alanine and serine residues, respectively. These mutations cause defects in the nuclear import of the protein and are implicated in retinitis pigmentosa type 26.^36,37^ In order to observe what changes in the physicochemical properties of SLiM residues are unfavourable, the frequencies of changes of these properties caused by mutations were compared between disease-related missense mutations and neutral missense mutations (see Methods) (ESI,† Fig. S1). Compared to the neutral missense mutations from the 1000GP dataset, inherited disease mutations (OMIM dataset) changed the amino acid properties of SLiMs more frequently, consistently across all types of properties in comparison (charge, hydropathy, polarity, volume, chemical characteristics, hydrogen donor/acceptor availability). For three of the six properties in comparison, OMIM mutations caused changes in the physicochemical properties of SLiM residues significantly more frequently than the 1000GP mutations: hydropathy (76% of OMIM mutations; 53% of 1000GP mutations; *p* = 0.024); hydrogen donor/acceptor availability (76% of OMIM mutations; 62% of 1000GP mutations; *p* = 0.039); and side chain chemistry (85% of OMIM mutations and 70% of 1000GP mutations; *p* = 0.015). On the other hand, no significant differences were observed between the COSMIC dataset and the 1000GP dataset in terms of the frequency of transitions in the physicochemical properties of SLiM residues. This result suggests that inherited disease mutations from the OMIM dataset have a more evident impact on the physicochemical properties of SLiM residues than cancer related mutations from the COSMIC dataset.

To further elucidate the specific kinds of unfavourable changes in the physicochemical properties of SLiM residues, the frequencies of each type of transitions were compared between the disease-related and neutral mutation datasets. For each class of physicochemical properties (charge, hydropathy, polarity, volume, chemical characteristics, hydrogen donor/acceptor availability), amino acids were grouped according to subclasses of each property (for example, based on the hydropathy properties, amino acids were grouped into three subclasses: hydrophobic, hydrophilic, and neutral) (see Methods). Between the OMIM and the 1000GP datasets, none of the transitions among hydropathy properties (hydrophobic, hydrophilic, neutral) and none of the transitions among polarity properties (non-polar and polar) were significantly different. In terms of transitions among charge properties (positively charged, negatively charged, uncharged), OMIM mutations significantly more frequently substituted uncharged residues with positively charged residues (*p* = 0.008). When amino acids were grouped based on their volumes, OMIM mutations changed very small residues in SLiMs to very large residues significantly more often than the 1000GP mutations (*p* = 0.002). Interestingly, the OMIM mutations substituted the wild type residues with mutant residues that have a larger volume more often than the 1000GP mutations (57% of the OMIM mutations and 41% of the 1000GP mutations caused an increase in the volume of the SLiM residues; *p* = 0.035). Furthermore, the OMIM dataset was enriched for mutations that changed SLiM residues which had neither hydrogen donor nor hydrogen acceptor atoms to residues with hydrogen donor atoms (*p* = 0.009). Finally, the OMIM dataset was enriched for transitions in the side chain chemistry such as hydroxyl to aromatic (*p* = 0.009), basic to hydroxyl (*p* = 0.025), and aliphatic to basic (*p* = 0.022) (ESI,† Fig. S2). Between the COSMIC dataset and the 1000GP dataset, as observed for the comparison of the OMIM dataset and the 1000GP dataset, significant differences were observed for transitions from very small to very large residues (*p* = 0.025) and from hydroxyl to aromatic side chain chemistries (*p* = 0.04). However, for the rest of the transitions, no significantly different transitions of physicochemical properties of SLiM residues were observed between the COSMIC dataset and the 1000GP dataset (ESI,† Fig. S3).

### Recurrently mutated SLiMs in human diseases

**Fig. 3:**
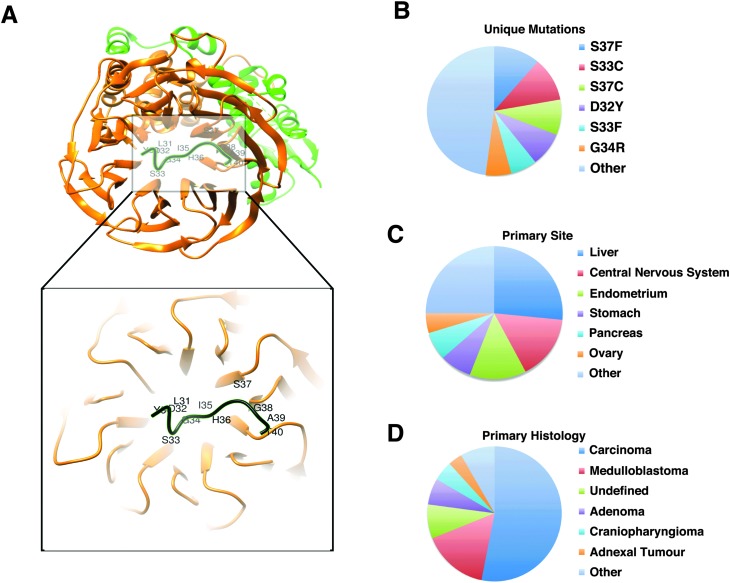
Phospho-degron motif of β-catenin. (A) Structure (PDB:1P22) of the β-TrCP1-Skp1-β-catenin complex^44^ generated with Chimera.^45^ Green: Skp1; orange: beta-TrCP1; black: β-catenin phospho-degron motif (DSGx{2,3}[ST], _32_D_p_SGIH_p_S_37_). The degron motif binds the WD40 repeat domain of β-TrCP1. (B–D) Classification of 1709 entries in the COSMIC dataset that reports 33 unique missense mutations (B) derived from 1692 different samples taken from 27 unique primary sites (C) with 26 unique primary histology descriptions (D).

According to the available disease-related missense mutation datasets, the most recurrently mutated experimentally validated SLiM is the conserved proteasomal degradation motif (“degron”) in the highly disordered N-terminal region of β-catenin (Fig. 3A). This motif (DEG_SCF_TRCP1_1, _32_D_P_SGIH_P_S_37_) mediates binding to the WD40 repeat domain of the beta-TRCP subunit of the SCF-betaTRCP E3 ubiquitin ligase complex (Fig. 3B). In the COSMIC dataset, there are 1709 mutation entries for this motif derived from 1692 unique samples based on 256 different publications. Each of the six positions of the motif contains at least one mutation (a total of 33 unique mutations). These 1692 samples are from 27 primary tumour sites (454 samples from the liver and 271 samples from the central nervous system as the top two primary sites) with a diverse set of 26 primary histology descriptions (908 of the samples classified as carcinoma and 269 of them classified as medulloblastoma as the top two primary histology types) (Fig. 3C). Of note, all of the most commonly occurring mutations of this SLiM (*D32Y*, *S33F*, *S33C*, *G34R*, *S37F*, *S37C*) occur on functionally important residues and are categorised as ‘motif-breaking’ mutations. Other examples of recurrently mutated experimentally validated SLiMs include Cellular Tumour Antigen p53's nuclear localisation signal (_305_KRALPNNTSSSPQPKKKPL_323_),^38^ the 14-3-3 binding motif of Raf1 (_256_RST_p_STP_261_),^39^ and the VHL degron motif of endothelial PAS domain containing protein 1 (HIF2α) (_529_LAPYIOHPMDGEDFQR_542_)^40^ (ESI,† Table S9).

### Mutations in the predicted SLiMs

The human proteome has the capacity to contain millions of SLiM instances.^41^ Manual annotation of SLiMs is an accurate but slow process, and therefore, should be supported by computational prediction tools. SLiM prediction is a computationally challenging problem because they are short and often degenerately defined (allowing physico-chemically similar substitutions at certain positions). So, particularly for the SLiM classes that have high occurrence probability, a regular expression search in the proteome results in many false-positive motif instances. However, predictions can be improved by filtering hits in inaccessible regions and by retaining only well conserved instances, which are strategies utilised by the SLiMSearch^42^ and SlimPrints^43^ motif discovery tools.

**Fig. 4:**
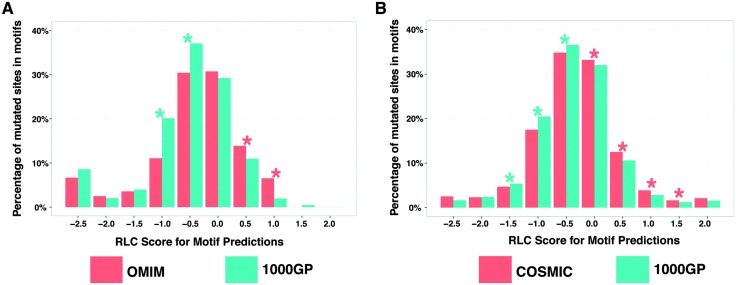
Mutation enrichment analysis in predicted SLiM instances. Frequencies of mutated sites within predicted SLiM instances (IUPred = 0.5) at different relative local conservation score intervals are compared. RLC scores range from –2.5 to 2. SLiMs that are relatively more conserved than the surrounding regions have a RLC score above zero. (A) Comparison between OMIM and 1000GP (green stars represent enrichment of 1000GP mutations; red stars represent enrichment of OMIM mutations) and (B) COSMIC and 1000GP (green stars represent enrichment of 1000GP mutations; red stars represent enrichment of COSMIC mutations).

Based on our prior results, if a candidate SLiM in a disordered region is truly functional, a mutation in the SLiM is more likely to be disease-associated. Likewise, if a mutation in a predicted SLiM contributes to disease, the SLiM is more likely to be functional than a random peptide that matches the SLiM pattern. A disease-related mutation in a SLiM may disrupt a protein–protein interaction, which may be important for signaling and regulation. Based on this logic, we hypothesised that if a given list of predicted SLiMs contains a reasonable number of truly functional motifs, we should observe an enrichment of disease-related mutations in those SLiMs compared to the background. For this purpose, we predicted SLiMs in disordered segments (IUPred score > 0.5) of the human proteome and compared the level of enrichment/depletion of disease-related mutations against the background (Fig. 4A and B).

Missense mutations from the 1000GP dataset were significantly more frequently found in predicted SLiMs that had poor relative conservation scores (RLC score < 0). On the other hand, a significant enrichment of disease-related mutations was observed for the predicted SLiMs that had positive relative local conservation scores (RLC > 0). This result adds support to our previous findings that disease-related missense mutations occur more frequently in SLiMs in the IDRs than neutral missense mutations. Furthermore, in this set of predicted SLiM instances using stringent disorder and RLC scores (see Methods) (ESI,† Table S10), compared to experimentally validated SLiMs, there were ∼63 fold more candidate SLiM instances with mutations from the COSMIC dataset (18 990 predicted SLiM instances with mutations) and ∼13 fold more candidate SLiM instances with mutations from the OMIM dataset (403 predicted SLiM instances with mutations). These predicted SLiM instances containing disease-related mutations can serve as a strong list of candidates, which may be of interest to other researchers for follow-up studies (ESI,† Table S11).

### Mutated SLiMs in protein–protein interaction (PPI) networks

**Fig. 5:**
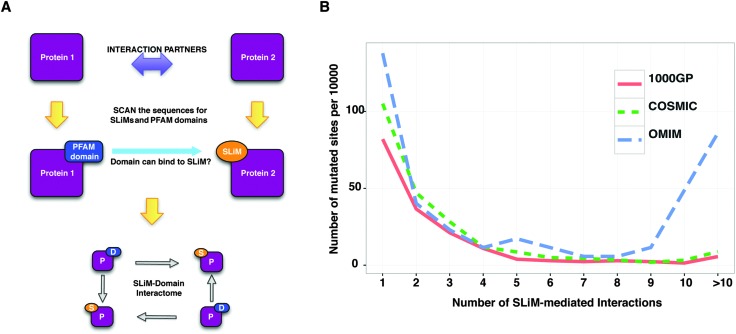
Analysis of mutations in the predicted SLiM–domain interaction network. (A) Pipeline of SLiM–domain interaction network construction. (B) Distribution of the number of interactions (*x* axis) *versus* number of mutated sites (normalized per 10 000 mutated sites – *y* axis) (see Methods). The relative frequency distributions (number of interactions per 10 000 mutated sites) of each mutation dataset are plotted using a blue dashed line (the OMIM dataset), a green dashed line (the COSMIC dataset), and a straight red line (the 1000GP dataset).

In scale-free networks such as PPI networks, defects in the hubs have more deleterious effects on the network compared to defects in non-hubs.^46^ A study has demonstrated that, for yeast, deletion of hub proteins imposes a higher risk of lethality to the organism.^47^ Thus, the more interactions a protein has, the worse the consequences for the network will be when the protein loses its interactions with the surrounding proteins. SLiMs are important mediators of protein–protein interactions and SLiM mediated interactions can be lost due to mutations in the SLiMs. For instance, mutations in the 14-3-3-binding motif of Raf1 abrogate its interaction with 14-3-3 proteins in Noonan and Leopard syndromes.^48^ Although examples exist of diseases caused by the known loss of protein–protein interactions due to mutations in the SLiMs, we wanted to observe whether there is a trend at a proteome-wide scale such that the more interactions a SLiM mediates, the higher is its likelihood to be associated to disease. If so, disease-related mutations should impact more SLiM mediated interactions than should neutral mutations. In order to make a comparison, using the predicted list of SLiMs, a motif-mediated PPI network was constructed (see Methods) (Fig. 5A) (ESI,† Table S12). Then, for each mutated site in the disordered regions of the proteome (IUPred = 0.5), the total number of protein–protein interactions mediated by the predicted SLiMs that overlap the mutated site was counted. The number of interactions for the mutated sites, which do not overlap any of the predicted SLiMs involved in the interaction network, was counted as zero (ESI,† Tables S13–S15). The number of SLiM-mediated interactions impacted by disease-related mutations was higher than that of neutral mutations (Fig. 5B) for both COSMIC (*p* < 1.276 × 10^–12^, Wilcoxon rank-sum test) and OMIM (*p* < 1.628 × 10^–7^, Wilcoxon rank-sum test). This result suggests that the number of interactions a SLiM mediates influences the likelihood that a mutation in a SLiM is disease-related.

### Pathways enriched with disease-related SLiM-containing proteins

**Fig. 6:**
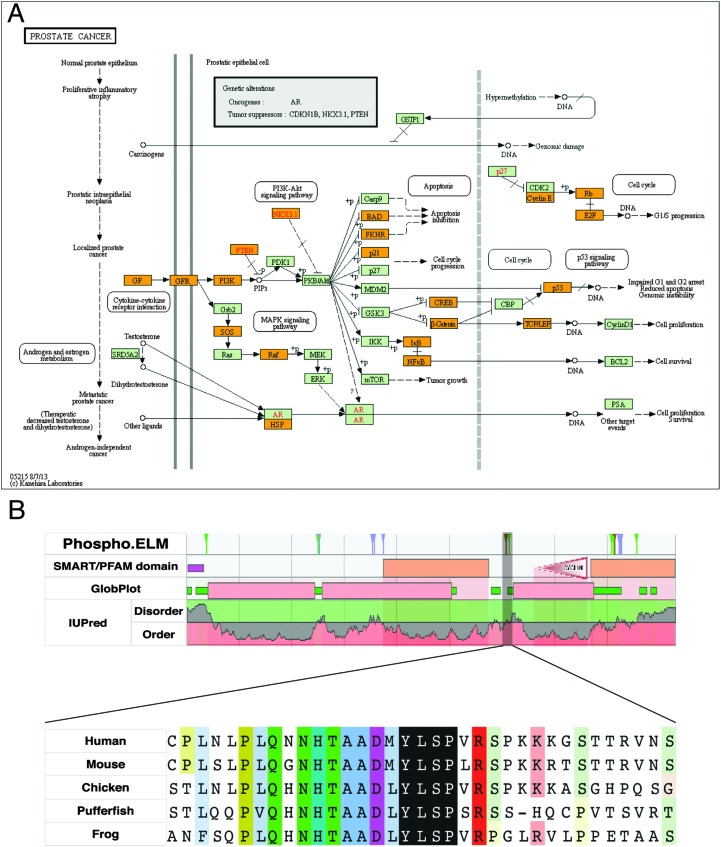
Analysis of predicted SLiMs with motif-breaking mutations in the KEGG human prostate cancer pathway. (A) KEGG human prostate cancer pathway (KEGG id: hsa05215^53^). Proteins highlighted with orange in the pathway contain at least one predicted SLiM that has a motif-breaking mutation (COSMIC). (B) Sequence features of retinoblastoma-associated protein-1 (RB1) (phosphorylation sites from Phospho.ELM,^54^ domain predictions from SMART^55^ and PFAM,^56^ and order–disorder profile predictions from GlobPlot^57^ and IUPred^58^). A potentially functional SH2-binding motif (_606p_YLSP_609_) with a multiple sequence alignment of orthologs from representative model organisms is highlighted in black. The alignment is generated using ClustalW^59^ and visualized using JalView.^60^

Some cellular pathways may be more dependent on motif functionality than others. In order to observe such differences between pathways, we looked for the pathways that are most enriched with predicted SLiMs that contain motif-breaking mutations from the COSMIC dataset (Table 4). Proteins containing disease-related SLiMs were most enriched in the ‘KEGG human prostate cancer pathway’ (Fig. 6A). In this pathway, 26 proteins had at least one predicted SLiM with a motif-breaking mutation (ESI,† Table S16). A manual literature search revealed that, of these 26 proteins, seven proteins had at least one predicted disease-related SLiM that was experimentally validated and already annotated in the ELM resource; nine proteins had at least one predicted disease-related SLiM with experimental validation, but was not annotated in the ELM resource; five proteins had at least one predicted SLiM that neither had experimental validation nor was annotated in the ELM resource but showed promising evidence of functionality; and five of them lacked mutated predicted SLiMs with any experimental validation or promising evidence that suggested functionality of the SLiM (see Methods).

**Table 4:** DAVID–KEGG pathway enrichment analysis results (FDR < 0.05)

KEGG pathway	Number of proteins	*p*-value	FDR
hsa05215: Prostate cancer	26	2.54 × 10^–6^	0.003
hsa05213: Endometrial cancer	18	1.17 × 10^–5^	0.014
hsa04510: Focal adhesion	42	1.68 × 10^–5^	0.020
hsa03040: Spliceosome	30	2.98 × 10^–5^	0.036

For some of the predicted SLiMs with motif-breaking mutations in the ‘KEGG human prostate cancer pathway’, supporting experimental evidence of function can already be found in the literature and annotated in the ELM resource. For instance, the stability of IκBα, an inhibitor of NFκB, is regulated *via* a phospho-degron motif (_31_D_p_SGLD_p_S_36_). Regulation of NFκB activation *via* modification of the stability of IκBα is crucial as NFκB signals the transcription of genes involved in a variety of cellular processes including immune response, inflammation, differentiation, and apoptosis.^49^ In patients with anhidrotic ectodermal dysplasia with T cell immunodeficiency, a *S32I* mutation in IκBα protein has been found.^50^ This mutation disrupts the phosphorylation of the IκBα degron motif; thus the protein cannot be degraded and ultimately NFκB cannot be activated. Another motif-breaking mutation (*D31N*) was found in a breast cancer sample (COSMIC). This mutation may impair regulation of NFκB, and may thus be detrimental to a variety of cellular processes. For some of the SLiMs that were not annotated in the ELM resource, we could still find experimental validation in the literature for their functionality. For instance, human TCF3, a transcription factor that acts as an activator of Wnt signaling in the presence of β-catenin, contains a C-terminal-binding protein 1 (CtBP)-binding motif (_502_PLSLT_506_). In the absence of β-catenin, TCF3 binds to CtBP co-repressor and it acts as a transcriptional repressor.^51,52^ A motif-breaking S504P mutation, found in a large intestine carcinoma sample (COSMIC), may be responsible for the loss of regulation imposed by CtBP for the repressor activity of TCF3.

Some mutated SLiMs in this pathway are potentially functional, but require further experiments to characterise the motif's functionality and the functional impact of a mutation in the motif. For instance, retinoblastoma-associated protein (RB1) has a predicted SH2 domain-binding site (_606p_YLSP_609_), which is conserved in a disordered region of the RB1 protein (Fig. 6B). There are two cancer-associated motif-breaking mutations in this motif: *Y606C* and *L607P* (COSMIC). As a complementary evidence for the functionality of this putative motif, RB1's Y606 is a known phosphorylation site in both humans^61^ and mice (corresponding phosphorylation site Y599).^62^

Moreover, there are several known SH2 domain-containing binding partners of RB1: tyrosine-protein kinase ABL1,^63,64^ tyrosine-protein kinase FRK,^65^ and signal transducer and activator of transcription 3 (STAT3).^66^ Among these proteins, STAT3 is known to directly bind to RB1 on DNA.^66^ Cumulatively, the available evidence suggests that this motif is a promising SH2-binding site that might be important for the regulatory functions of the RB1 protein.

Taken together, analysis of disease-related motif-breaking mutations in predicted SLiMs can lead us to potentially functional SLiMs. This in turn can improve our understanding of a protein's functionality in disease pathways. Of note, our analysis of mutated SLiMs in ‘KEGG human prostate cancer pathway’ suggests that the combinatorial impact of SLiM mutations could be extensive if they simultaneously malfunctioned. This finding underlies the necessity to understand the defects in SLiM functionality to better understand disease pathways.

## Discussion

### Structure-centric analysis of mutations

The now obsolete dogma of structural biology, ‘structure determines the function of a protein’, has historically biased the analyses of the impact of disease-related mutations on proteins toward folded globular domains. Researchers have tried to explain how mutations impact the properties of proteins that contribute to structural order of proteins. Similarly, algorithms that are designed to classify mutations based on their impact also have carried this bias for structured proteins.^14,67^ In molecular recognition, while ordered proteins are used mostly for catalysis and associated enzymatic processes, disordered proteins are mainly used for signaling and regulation.^11,68^ Cancer arises from alterations preferentially in the cellular signaling pathways.^69^ Such alterations can occur due to mis-recognition- or mis-signaling-based defects in IDPs.^23,70^ It has been postulated that point mutations in IDRs may disrupt SLiMs and contribute to mis-recognition- or mis-signaling-based diseases.^31,71^ In fact, the diverse functionality conferred by SLiMs onto IDRs is concomitantly impaired in a diverse set of human diseases as a result of mutations. For instance, the most well studied SLiM with cancer-associated mutations, the phospho-degron DSGxxS motif of β-catenin (_32_D_p_SGIH_p_S_37_), is required for the regulation of the stability of β-catenin, which is a key protein of the Wnt signaling pathway and is responsible for activation of Wnt-responsive genes for regulation of cell adhesion.^72^ Mutations in this phospho-degron motif lead to accumulation of β-catenin, resulting in constitutive activation of Wnt-responsive genes, which can drive various types of cancers.^73,74^ Other ways in which SLiM mutations contribute to disease include the following: altering the sub-cellular localisation of the protein (*e.g.* the ciliary trafficking motif of rhodopsin is mutated in autosomal dominant retinitis pigmentosa^75–77^); defective proteolytic cleavage (*e.g.* furin cleavage site of the insulin receptor is mutated in insulin resistant diabetes^78^); and/or impairing post-translational modification sites (*e.g.* mutation of the sumoylation site of microphthalmia-associated transcription factor (MITF) causes a five-fold increase in the risk of developing melanoma and renal cell carcinoma^79,80^) (see ESI,† S22 for more examples of SLiM functionality disrupted in diseases). Thus, bioinformatics tools that classify the functional impact of mutations should take into account the fact that mutations may impair the functions of proteins without impairing their structural properties such as folding. The proteome-wide analysis of mutated SLiMs presented in this study stresses that these occurrences are not isolated events and, as has been demonstrated before for post-translational modification sites,^15,16^ loss of SLiM functionality due to mutations can be a prevalent molecular mechanism that may be used to explain the underlying causes of human diseases.

### Implications for SLiM prediction

As argued above, the tools that predict the functional impact of mutations can/should benefit from understanding linear motif biology. In the same way, SLiM prediction tools can benefit from analyses of mutations in motifs. SLiM prediction algorithms have utilised a variety of parameters to improve prediction accuracy. These parameters include intrinsic disorder,^26^ evolutionary conservation,^43^ surface accessibility,^81^ protein–protein interactions,^82^ and GO term enrichment.^83^ In this study, when compared to neutral mutations, an enrichment of disease-related mutations was observed for the experimentally validated SLiMs in disordered regions. Similar results could be reproduced for SLiMs predicted in relatively conserved segments of disordered regions. These results suggest that analyses of mutations in the IDRs could suggest the presence of potentially functional SLiMs. Therefore, mutation analysis should be incorporated into prediction pipelines in order to improve confidence in the predictions.

Moreover, we have observed that the inherited disease-related mutations tend to occur on functionally important residues and break the defined pattern of experimentally validated SLiMs more often than neutral mutations. This finding suggests that, as a complementary evidence to evolutionary conservation, the distribution of the disease-causing and neutral missense mutations within SLiMs could be used to re-define, fine-tune, and improve the existing regular expressions that are used to define SLiM classes.

### Implications for drug design and treatment strategies

SLiMs have been studied in the context of deregulated expression of IDPs^84^ and in the context of infectious diseases caused by pathogens abusing SLiMs.^31,85^ Moreover, molecular compounds and drugs designed to target SLiM-mediated interactions have shown promising results for targeted treatment strategies.^31,86–89^ In this work, by analysing the impact of disease-related mutations on SLiMs, we have explored an additional important aspect that emphasises the therapeutic importance of SLiMs.

SLiMs can be deleterious to cells if used aberrantly, for instance, in the context of deregulated expression of IDPs.^84^ Amplified human oncoproteins are enriched with SLiMs and IDRs, and they are often involved in protein–protein interactions.^90^ As an illustration, over-expression of the murine double minute 2 (MDM2) protein, an E3 ubiquitin ligase, causes a decrease in the apoptotic activities of p53 and promotes tumourigenesis,^91^ because binding of MDM2 to a FxxxWxxL motif in p53 promotes the proteasomal degradation of p53.^92^ Another aspect of SLiMs that has highlighted their therapeutic relevance is the fact that SLiM-binding pockets are targets of bacterial, fungal, or viral pathogens.^31,85^ Through SLiM mimicry, pathogens gain access to cellular signaling and regulation pathways of the host, and thus exploit SLiM functionality to invade the host organism and create an environment that allows the pathogens to replicate and proliferate.^31,85^ For instance, a variety of DNA viruses replicate their genomes by utilising the retinoblastoma-associated protein-binding motifs (LxCxE) to force the host cell cycle to enter the S phase and activate the DNA replication machinery.^93^ In short, imbalanced expression of IDPs containing SLiMs and pathogenic mimicry of the SLiMs of the host cell illustrate two important aspects of the therapeutic relevance of SLiMs.

In this work, with an analysis of disease-related mutations in the experimentally validated and predicted SLiMs, we have explored a third aspect of SLiMs that emphasises their therapeutic importance. SLiM-mediated interaction interfaces have already begun to serve as non-classical targets for drug development efforts.^31^ Currently, two of the most promising drugs designed to target SLiM-mediated interactions are Nutlins (competing for binding to p53-binding site on MDM2) and Cilengitide (mimicking integrin-binding RGD peptides). Nutlins are already in clinical trials for retinoblastoma^86^ and liposarcoma,^87^ and Cilengitide has entered Phase III clinical trials for glioblastomas.^88^ Considering the successfully developed drugs that specifically target SLiM-mediated interactions, this may be a potentially high-promising avenue of investigation. Our analysis of mutations in SLiMs in the context of PPI networks suggests that there are many more potential targets within the proteome. Treatment strategies involving drugs that can target such interactions will possibly show an increase in the near future.

## Conclusions

We observed significant differences in the distribution of mutations in SLiMs between datasets of disease-related and neutral mutations. In particular, an enrichment of disease-related mutations in SLiMs compared to the background for both experimentally validated and predicted SLiMs was observed. These studies have allowed us to compile the most comprehensive list of disease-related SLiMs. When analysing the functional impact of mutations on proteins, the presence of SLiMs in the protein sequence should not be neglected. As more and more SLiMs are discovered and more genomes are sequenced, we will have a clearer picture of the roles of SLiMs in human diseases. In the next decade, an increase is expected in the number of studies that will reveal different mechanisms of how SLiMs are associated with human diseases and different treatment strategies.

## Methods

### Datasets

#### Protein sequences

UniProt Reference human proteome was downloaded (July 2012). Using the protocol described for the SLiMSearch motif prediction tool,^42^ 19 991 protein sequences, for which enough number of orthologs could be detected to calculate a multiple alignment, were kept (ESI,† Table S5).

#### Experimentally validated SLiMs

The Eukaryotic Linear Motif (ELM) resource^28^ is a collection of manually annotated, experimentally validated SLiMs curated from the literature for eukaryotic species. SLiM instances and classes annotated by the ELM resource were downloaded (October 2013). Only instances that are experimentally proven to be functional (annotated as ‘True Positive’) for *Homo sapiens* were kept. This set of SLiM instances comprised 1262 individual instances categorised into 161 classes of SLiMs in a total of 726 proteins (ESI,† Table S4).

#### Mutation datasets

Inherited disease mutations in humans are from UniProt annotations^34^ of Online Mendelian Inheritance in Man (OMIM)^35^ mutations with 1941 proteins containing at least one disease-associated mutation at 19 630 unique sites (ESI,† Table S1). This dataset consists of experimentally validated missense mutations that contribute to inherited diseases, so it serves as a high quality dataset. Inherited disease mutations were downloaded from UniProt (; http://www.uniprot.org/docs/humsavar.txt). Only mutations that were associated to ‘Disease’ were kept. ‘Unclassified’ mutations or ‘Polymorphisms’ were excluded.

The second disease-related missense mutation dataset is downloaded from the Catalog of Somatic Mutations in Cancer (COSMIC).^32^ Missense mutations in the COSMIC dataset are all derived from tumour samples. However, mutations found in tumour samples are not always proven to contribute to cancer.^94^ So, the COSMIC dataset is larger, but, in terms of experimental evidence for each reported mutation, has lower quality than the OMIM dataset. Somatic cancer-associated missense mutations of the COSMIC database version 66 were exported using COSMICMart (; http://cancer.sanger.ac.uk/biomart/martview/), for genes that were mapped to UniProt accession numbers. The COSMIC dataset consists of 13 941 proteins with 440 266 unique mutated sites (ESI,† Table S2).

We used the 1000GP dataset as the control dataset to understand the background distribution of neutral missense mutations in the proteome. This dataset consisted of 207 720 mutated sites in 12 755 proteins (ESI,† Table S3). The functional impact of the missense mutations from the 1000GP dataset is individually weak, but the collective effect of multiple mutations may have a bigger impact.^95^ Still, the majority of 1000GP mutations are polymorphisms with an allele frequency of more than 1%.^33^ Thus the majority, if not all, of the mutations from the 1000GP dataset are presumably commonly found variants in the population. On the other hand, mutations reported from disease-related mutation datasets are either known to be causal for disease or are found in disease samples (*e.g.* tumour tissue sequencing data from the COSMIC database). Therefore, as the 1000GP dataset is more likely to contain neutral mutations, it serves as a good control dataset to compare with disease-related mutation datasets. Neutral missense mutations, *i.e.* polymorphisms, published by the 1000 Genome Project Consortium were used based on the ID mapping to UniProt accession numbers in the SQL dump file generated by the SNPdbe database.^96^

#### PPI dataset

The non-redundant set of human protein–protein interactions was downloaded from iRefWeb (November 2013),^97^ a meta-database of protein–protein interactions that combines data from various PPI databases such as BIND,^98^ BioGRID,^99^ CORUM,^100^ DIP,^101^ IntAct,^102^ HPRD,^103^ MINT,^104^ MPact,^105^ MPPI,^106^ and OPHID.^107^ Only binary interactions of those proteins that had UniProt accession numbers were kept (ESI,† Table S17).

### Predictions

#### Disorder score prediction

Residue-based disorder tendencies of proteins were predicted using IUPred binaries^58,108^ using the default profile ‘LONG’ considering sequential neighbourhood of 100 residues (ESI,† Table S18). IUPred disorder scores above 0.5 denote regions of the proteins that have 95% likelihood to be disordered.

#### Relative local conservation (RLC) score prediction

Per-residue based relative local conservation scores (RLC) were calculated using the SLiMSearch motif discovery tool^42^ (ESI,† Table S19). RLC scores above zero denote regions of the proteins that are more conserved than the surrounding regions. RLC scores below zero denote regions of the proteins that are less conserved than the surrounding regions.

#### Motif prediction

SLiM instances for all proteins (19 991 protein sequences in the sequence dataset) were predicted by performing a regular expression search on the protein sequences using the motif definitions of 202 SLiM classes. Each individual SLiM instance was assigned start and end coordinates with respect to the matched sequence segment of the protein. Also, each SLiM instance was assigned disorder and RLC scores by averaging the corresponding scores of each residue of the SLiM instance. For filtering the candidate SLiMs, an IUPred disorder score cut-off of 0.5 was applied.^58^ As a second filtering score, based on the results of the mutation enrichment analysis in experimentally defined SLiMs, a stringent RLC score cut-off of 0.5 was chosen. This set of predicted SLiMs consists of 101 630 predicted SLiM instances from 177 SLiM classes in 10 243 proteins (ESI,† Table S10). These SLiMs take up 575 197 residues (5.2%) of the proteome, which is ∼68 fold more than the experimentally validated SLiMs.

#### Protein domain prediction

Protein domains were detected by scanning the protein sequences using the HMMER (v3.0) toolset^109^ with the PFAM profile hidden Markov models (HMMs)^56^ (ESI,† Table S20).

#### SLiM-mediated interactome construction

Known pairs of PFAM domains and SLiM classes that can bind to each other were downloaded from the ELM resource (http://elm.eu.org/infos/browse_elm_interactiondomains.html) (ESI,† Table S21). All protein sequences were scanned for PFAM domains and SLiMs as described above. Each binary protein interaction in the PPI dataset was queried for known interacting PFAM domains and SLiM classes. An edge in the SLiM-mediated interactome was created for each pair of interacting proteins if one of the proteins contained a SLiM instance that can be recognised by a PFAM domain in its partner (Fig. 5A).

### Statistics

All the scripts for the analyses were written in Python 2.7.3 (http://www.python.org) and the statistics were calculated using R (; http://www.r-project.org/).^110^ The plots in Fig. 2, 4, 5B and ESI,† Fig. S1–S3 were drawn using the ggplot2 library (; http://ggplot2.org/).^111^

#### Enrichment analysis of mutated sites in experimentally validated SLiMs

A pairwise comparison of the distribution of missense mutations was carried out between (a) the OMIM dataset and the 1000GP dataset and (b) the COSMIC dataset and the 1000GP dataset. For each pairwise comparison, proteins that are not shared by the compared datasets were excluded. Moreover, proteins that do not contain any experimentally validated SLiM instances were also excluded. In order to avoid biases for well-studied SLiM instances, a mutated site was counted only once whether or not multiple mutations are reported for that site. The mutated sites and SLiM instances in the compared datasets were divided into two bins as ‘disordered’ or ‘ordered’. The classification was done based on the average IUPred disorder score of the SLiMs and the individual IUPred score of the mutated sites. SLiMs or mutated sites that have an IUPred score above 0.5 were categorised as ‘disordered’ and the rest were categorised as ‘ordered’. Mutated sites in ‘ordered’ or ‘disordered’ categories were further divided into two more categories as ‘within motif’ if the mutated site overlaps any of the SLiM instances and ‘outside motif’ if the mutated site does not overlap any of the SLiM instances. For each category of ‘disordered’ or ‘ordered’ regions, a 2 × 2 contingency table (two rows for the mutation datasets and two columns for the category of overlap with SLiMs, either ‘within motif’ or ‘outside motif’) was created. To test if there is a significant difference in the percentage of the mutated sites overlapping the SLiMs in the corresponding categories, a Fisher's exact test was applied using the contingency tables created specifically for the pairwise comparison of the mutation datasets.

#### Enrichment analysis of mutated sites in predicted SLiMs

A pairwise comparison of the distribution of the missense mutations was carried out between (a) the OMIM dataset and the 1000GP dataset and (b) the COSMIC dataset and the 1000GP dataset by considering only the shared proteins between the compared datasets as described for the ‘Enrichment Analysis of Mutated Sites in Experimentally Validated SLiMs’. Again, each mutated site was counted only once regardless of the number of unique mutations in that mutated site. The predicted SLiMs (see Methods – ‘Motif prediction’) and the mutated sites were grouped based on their relative local conservation (RLC) scores. For each of these groups, a 2 × 2 contingency table was created (two rows for the categorisation of mutated sites as ‘within motif’ and ‘outside motif’ and two columns for the two datasets that were compared) and a Fisher's exact test was applied to see if there is any significant difference between the compared datasets in terms of the frequency of the mutated sites overlapping the SLiMs.

#### Classification of unique mutations overlapping experimentally validated SLiMs

Each unique mutation overlapping the SLiMs from each mutation dataset is classified as ‘only motif-breaking’, ‘only motif-conserving’, and ‘both motif-breaking and motif-conserving’. A mutation is classified as ‘motif-breaking’ if the mutation changes the sequence of a SLiM instance in such a way that the regular expression pattern that defines the SLiM no longer matches the mutated sequence. On the other hand, if the mutated sequence still matches the regular expression pattern of the SLiM, the mutation is classified as ‘motif-conserving’. As the SLiM instances may be overlapping each other, some mutations may overlap multiple SLiM instances. Depending on the pattern of the different overlapping SLiMs, a mutation that overlaps both SLiMs may be classified as ‘motif-breaking’ for one SLiM instance or ‘motif-conserving’ for another SLiM instance. Such mutations are classified as ‘both motif-breaking and motif-conserving’. In order to test if there is a significant difference in the frequency of mutations to be classified as ‘motif-breaking’ in different mutation datasets, a pairwise comparison of mutation datasets was carried out. For each comparison (*e.g.* COSMIC *vs.* 1000GP), a 3 × 2 contingency table was created. The table consisted of two rows for the compared mutation datasets and three columns for the sizes of the mutually exclusive categories of mutations as described above. Fisher's exact test was applied to see if there was a significant difference between the datasets in terms of the frequency of mutations in different categories.

#### Analysis of the impact of mutations on the amino-acid properties of SLiMs

The twenty main amino acids found in the human proteome were classified for each of the six main physicochemical properties including charge, hydropathy, polarity, volume, chemical characteristics, and hydrogen donor/acceptor availability.^112^ For the ‘charge’ property, amino acids were grouped as positively charged (R, H, K), negatively charged (D, E), or uncharged (A, N, C, Q, G, I, L, M, F, P, S, T, W, Y, V). For the ‘hydropathy’ property, amino acids were grouped as hydrophobic (A, C, I, L, M, F, W, V), neutral (G, H, P, S, T, Y), or hydrophilic (R, N, D, Q, E, K). Based on their ‘polarity’, amino acids were grouped as polar (R, N, D, Q, E, H, K, S, T, Y) or non-polar (A, C, G, I, L, M, F, P, W, V). Based on their ‘volume’, amino acids were grouped as very small (A, G, S), small (N, D, C, P, T), medium (Q, E, H, V), large (R, I, L, K, M), or very large (F, W, Y). According to the chemical characteristics of the side chains, amino acids were grouped as aliphatic (A, G, I, L, P, V), aromatic (F, W, Y), sulfur (C, M), hydroxyl (S, T), basic (R, H, K), acidic (D, E), or amide (N, Q). Finally, based on the hydrogen donor/acceptor availability of atoms, the amino acids were grouped as donor (R, K, W), acceptor (D, E), donor and acceptor (N, Q, H, S, T, Y), or neither (A, C, G, I, L, M, F, P, V).

Firstly, the unique missense mutations that overlap the experimentally validated SLiMs were found for each missense mutation dataset (OMIM, COSMIC, and 1000GP datasets). Then, for each mutation, the physicochemical properties of the wild type and the mutant residues were determined. Based on the transitions between the amino acids and their properties from the wild type to the mutant, a (*N* by *N*) matrix of transition frequencies was calculated for each class of amino acid properties (charge, hydropathy, polarity, volume, chemical characteristics, and hydrogen donor/acceptor availability), where *N* is the number of sub-classes of the corresponding property. From these matrices, the frequencies of transitions were compared between the disease-related missense mutation datasets (OMIM and COSMIC) and the neutral mutation dataset (1000GP).

The percentage of mutations that cause a ‘change’ in a physicochemical property is calculated by the sum of the values outside of the main diagonal in the given matrix (where the row and the column are not defined by the same sub-class of the property) divided by the total sum of all the values in the matrix. Thus, for the comparison between the disease-related mutation dataset and the neutral mutation dataset, a 2 × 2 contingency table is created, where the rows are denoted by the compared mutation datasets and the columns are denoted by the frequency of SLiM mutations that change or do not change the corresponding physicochemical property. A Fisher's exact test is applied to find out if the disease-related mutation dataset has significantly more mutations that cause a change in the physicochemical properties of SLiM residues.

In order to find out if there are specifically unfavourable transitions between the sub-classes of physicochemical properties (for example, hydrophobic to hydrophilic transition causing a change in hydropathy), the calculated transition matrices were used again. This time, a Fisher's exact test was applied for each transition between every pair of sub-classes of each class of physicochemical properties. Let P denote an amino-acid property (*e.g.* hydropathy) and S1, S2,…,S*n* denote ‘*n*’ different sub-classes of a physicochemical property (*e.g.* S1 = hydrophobic, S2 = hydrophilic, S3 = neutral). For each transition from S*i* to S*j* (where 1 ⇐ *i*,*j* ⇐ *n*), a *p*-value was calculated to find out if the transition frequency from sub-class S*i* to sub-class S*j* is significantly different between different mutation datasets. For this, a 2 × 2 contingency table was created where the rows are the compared datasets and the columns denote (1) the number of mutations that cause transitions from S*i* to S*j* and (2) the total number of mutations that cause transitions from S*i* to every other sub-class except S*j*. A Fisher's exact test was applied on this contingency table to find out if there was a significant difference between mutation datasets for this type of transition of physicochemical properties in SLiM residues.

#### Analysis of mutations in the SLiM-mediated interactome

For proteins that contain a predicted SLiM instance, a SLiM-mediated interaction network was constructed as described in ‘SLiM-mediated interactome construction’. Based on this interactome, for each mutated site in disordered regions of the human proteome, the number of protein–protein interactions mediated by SLiMs that overlap the mutated site was counted. In order to account for the size differences between the mutation datasets, the number of mutated sites (per number of interactions) was divided by the dataset size (total number of mutated sites in the disordered regions) and multiplied by 10 000. A pairwise comparison of disease-related missense mutation datasets with the neutral missense mutation dataset (COSMIC *vs.* 1000GP, OMIM *vs.* 1000GP) was carried out. A Wilcoxon rank-sum test was used to find out if there is any significant difference in the number of SLiM-mediated interactions that are disrupted by disease-related mutations compared to neutral mutations.

#### Pathway enrichment analysis

SLiMs were predicted as described in ‘Motif prediction’. COSMIC mutations were mapped onto the SLiMs and mutations were classified as ‘motif-breaking’ or ‘motif-conserving’ mutations. Those proteins that contain a SLiM prediction with at least one motif-breaking mutation were further filtered for SLiM classes that have a motif occurrence probability below 0.01. UniProt accession numbers of proteins that contained predicted SLiM instances with a motif-breaking mutation were uploaded to the DAVID bioinformatics tools^113^ to retrieve the KEGG pathways^53^ that are most enriched for the uploaded proteins.
