# Supplementary material for: Proteome-wide analysis of human disease mutations in short linear motifs: neglected players in cancer?
Source: Mol Biosyst. 2014 Jul 24;10(10):2626–42. doi: 10.1039/c4mb00290c (PMC4306509; doi:10.1039/c4mb00290c)
Supplement: Supplementary file 1 [file MB-010-C4mb00290c-s001.pdf]

This journal is © The Royal Society of Chemistry 2014

SUPPLEMENTARY FIGURES 1-3

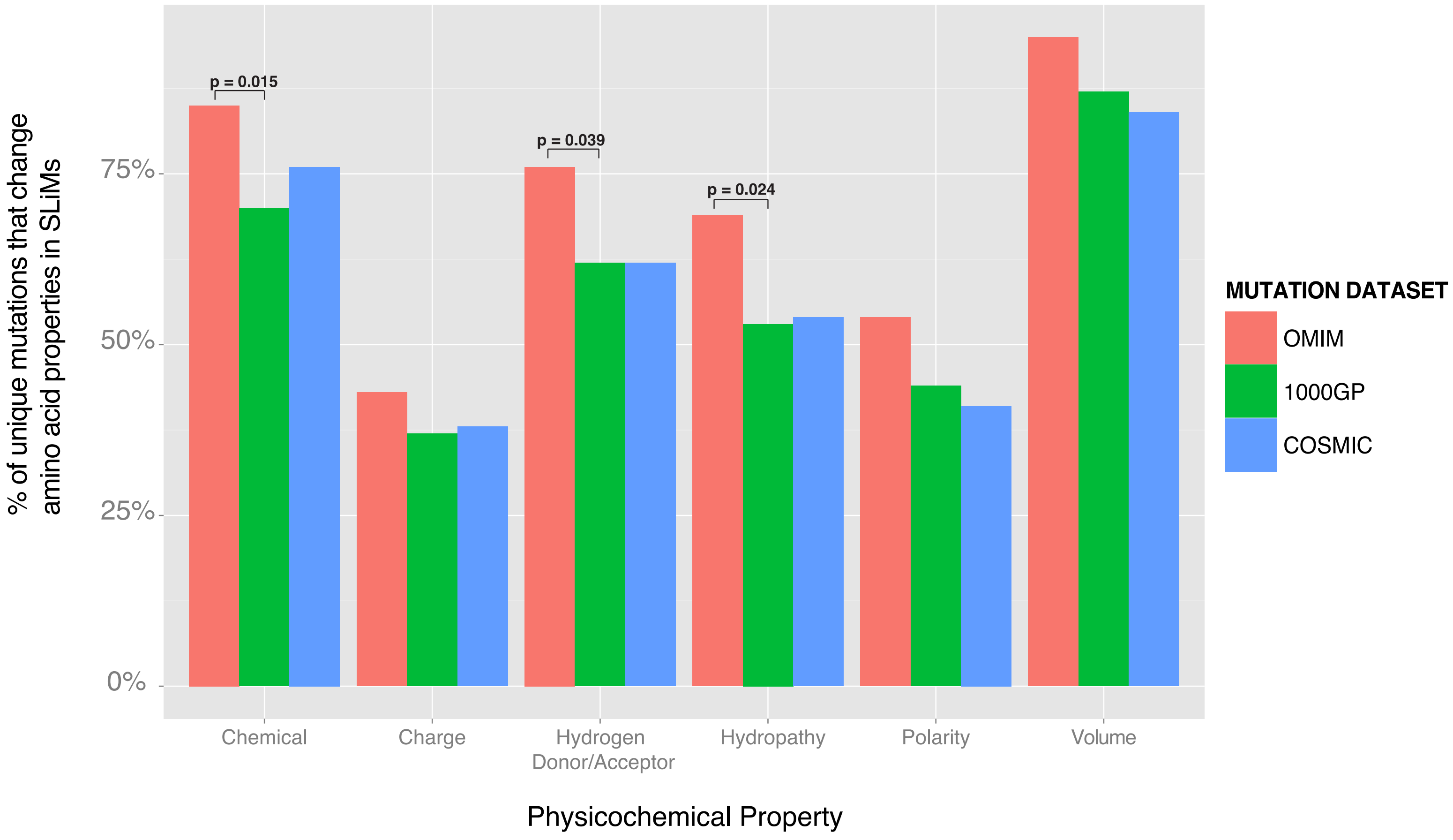

Supplementary figure 1: Comparison of the percentage of missense mutations in experimentally validated SLiMs that change the physicochemical properties of SLiM residues. Amino acids are grouped according to the following physicochemical properties: chemical (aliphatic, aromatic, sulphur, hydroxyl, basic, acidic, or amide); charge (positive charged, negative charged, or uncharged); hydrogen donor/acceptors (donor, acceptor, donor and acceptor, or neither); hydropathy (hydrophobic, hydrophilic, or neutral); polarity (polar or non-polar); volume (very small, small, medium, large, or very large).

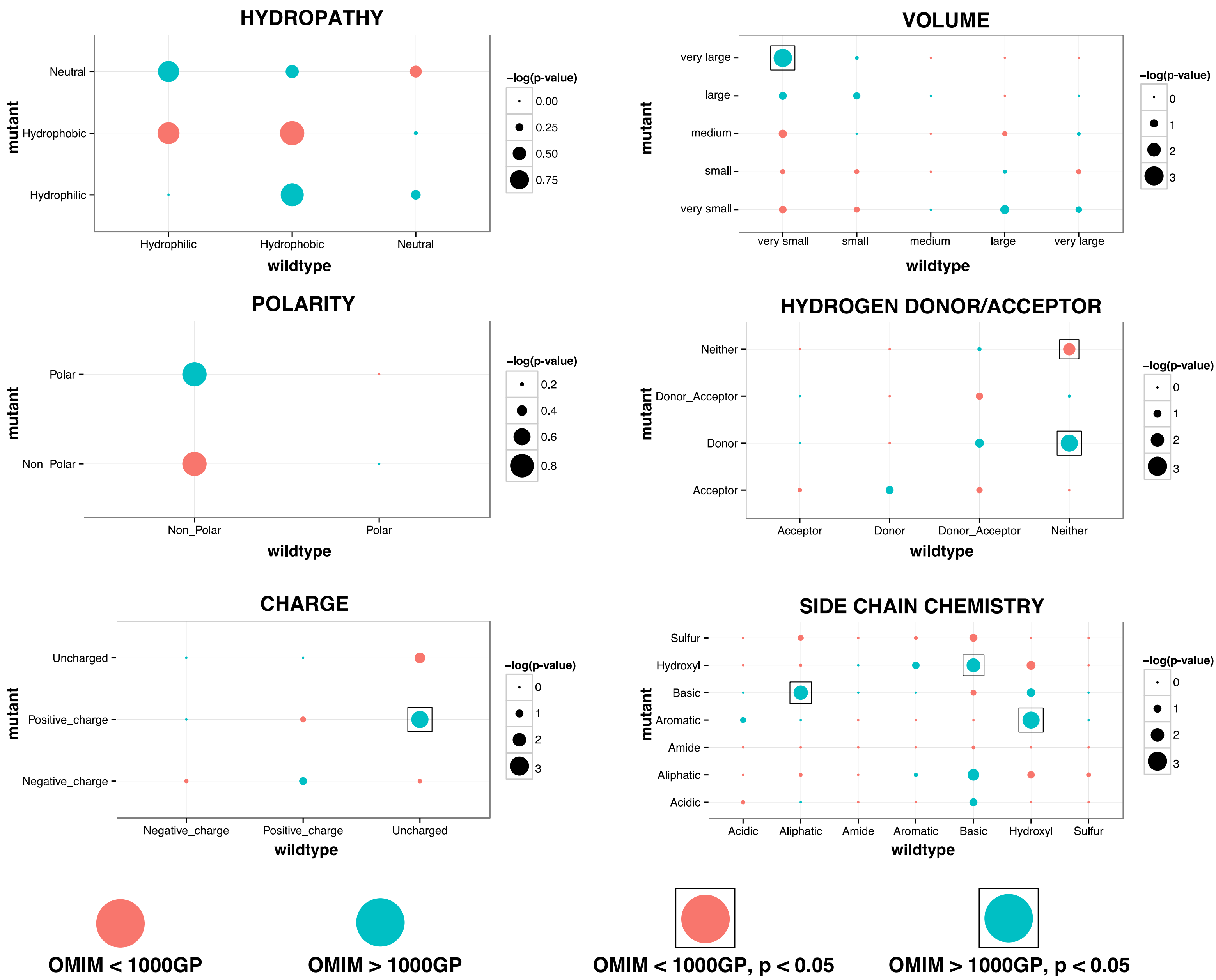

Supplementary figure 2: Comparison of the frequencies of the transitions in the physicochemical properties of experimentally validated SLiM residues caused by mutations overlapping the SLiMs. X-axis denotes the physicochemical property of the wild type residue and the Y-axis denotes the physicochemical property of the mutant residue. Circle sizes are proportional to the $-\log_{10}$(p-value). Red circles denote that ratio of mutations that cause the corresponding transition is higher in the 1000GP dataset compared to the OMIM dataset and vice versa for the green circles. Squares around the circles denote significant differences between the compared datasets.

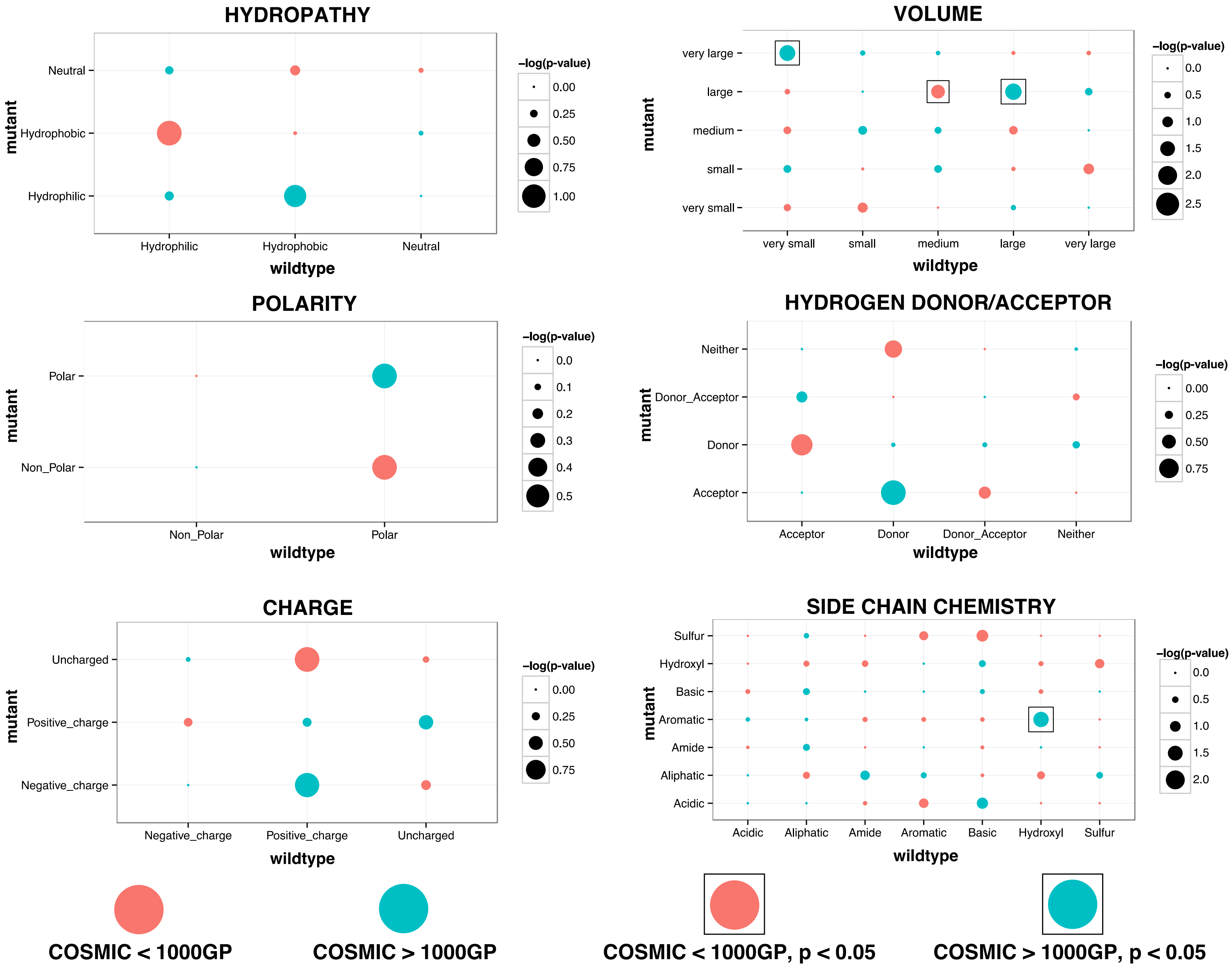

Supplementary figure 3: Comparison of the frequencies of the transitions in the physicochemical properties of experimentally validated SLiM residues caused by mutations overlapping the SLiMs. X-axis denotes the physicochemical property of the wild type residue and the Y-axis denotes the physicochemical property of the mutant residue. Circle sizes are proportional to the $-\log_{10}$(p-value). Red circles denote that ratio of mutations that cause the corresponding transition is higher in the 1000GP dataset compared to the COSMIC dataset and vice versa for the green circles. Squares around the circles denote significant differences between the compared datasets.
